# Concepts in Boolean network modeling: What do they all mean?

**DOI:** 10.1016/j.csbj.2020.03.001

**Published:** 2020-03-10

**Authors:** Julian D. Schwab, Silke D. Kühlwein, Nensi Ikonomi, Michael Kühl, Hans A. Kestler

**Affiliations:** a Institute of Medical Systems Biology, Ulm University, Albert-Einstein-Allee 11, 89081 Ulm, Germany; b Institute of Biochemistry and Molecular Biology, Ulm University, Albert-Einstein-Allee 11, 89081 Ulm, Germany

**Keywords:** Boolean network model, Simulation, Perturbation, Robustness, Phenotype, Drug screening

## Abstract

Boolean network models are one of the simplest models to study complex dynamic behavior in biological systems. They can be applied to unravel the mechanisms regulating the properties of the system or to identify promising intervention targets. Since its introduction by Stuart Kauffman in 1969 for describing gene regulatory networks, various biologically based networks and tools for their analysis were developed. Here, we summarize and explain the concepts for Boolean network modeling. We also present application examples and guidelines to work with and analyze Boolean network models.

## Graphical abstract

**Figure:**
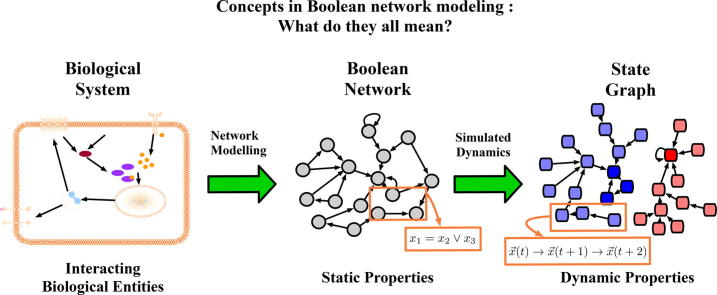

## 1 Introduction

**Fig. 1:**
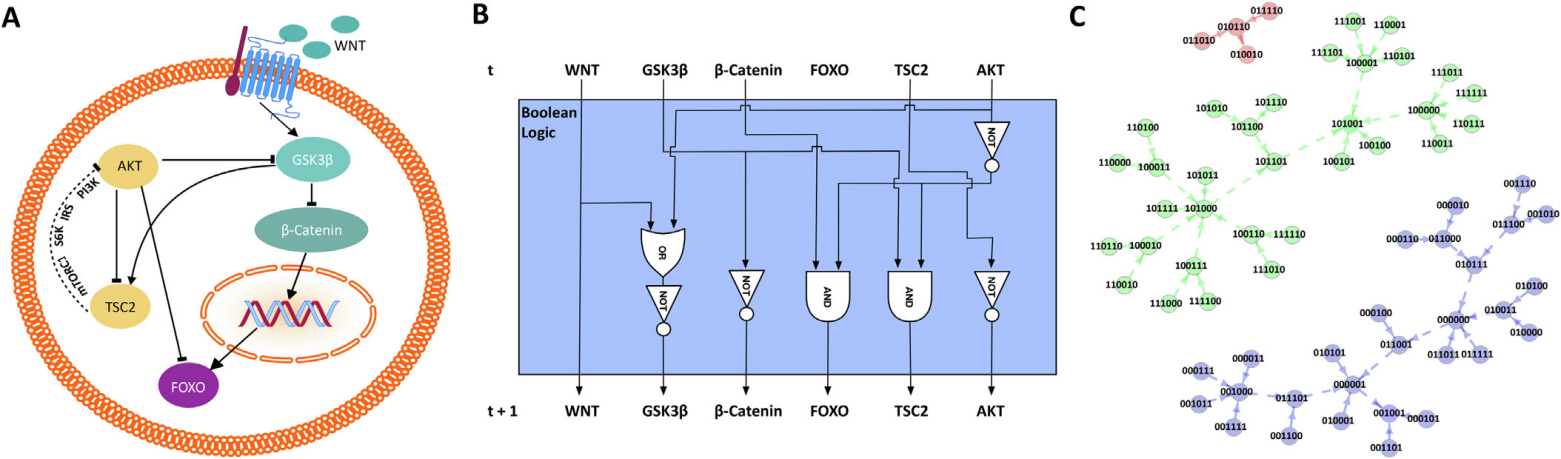
From biology to Boolean network models. Panel (A) displays one part of the FOXO cascade. The sketch-plot gives a static view on the different biological components and their interactions. However, dynamic properties of the system cannot be derived from this representation. (B) Shows the Boolean network model of the cascade in (A) depicted as logical circuit (blue box). Boolean functions are used to model the regulatory interactions between the different components. These functions are translated to a set of logic gates (AND/OR/NOT). The transition of each component from a certain time *t* to *t + 1* is evaluated in this circuit. (C) The complete dynamics of the given network are depicted as a directed graph. Each node shows one possible assignment of each component (here denoted as binary string). Arrows represent the transition from one state to its successor. The dynamics of the given example show three disjunct subparts of the graph which correspond to three different phenotypical patterns. (For interpretation of the references to colour in this figure legend, the reader is referred to the web version of this article.)

The development of diseases, aging, or even the maintenance of homeostasis are complex processes influenced by numerous factors [1]. Molecular studies of isolated interactions alone are no more sufficient to understand biology at a system level (Fig. 1A). Exemplarily, it is hard to judge if the crosstalk of multiple enhancers and silencers present at a promoter can transactivate transcription [2] or to evaluate feedback regulations in drug resistance as shown for AKT inhibitors [3], [4]. Therefore, the dynamic properties of biological networks have moved into focus [5] which can be assessed through mathematical models. Depending on the available information, dynamic models can be of a qualitative or quantitative nature [6]. Since quantitative models such as ordinary differential equation models require kinetic parameters, they are only feasible for small and well-investigated systems [7].

Boolean network (BN) models are one of the simplest dynamic models [8], [9]. In BN models, one implicitly assumes that all biological components are described by binary values and their interactions by Boolean regulatory functions [8], [9] (Fig. 1B). Simulation of Boolean networks gives insights into the dynamics of the respective system (Fig. 1C). Although simple in their composition, BN models have been applied to a wide range of processes from development [10] to aging [11]. Furthermore, they were used to uncover regulatory interactions leading to protein overexpression in cancer [12] or to screen for promising intervention strategies [13].

In this review, we summarize and explain the concepts of BN models and illustrate how this kind of model can be applied to address new biologically motivated hypotheses.

## 2 Boolean network models

BNs contain a set of variables $X=\left\{x_{1},x_{2},...,x_{n}\right\},{\;x}_{i}\in B$. Each of these variables represents one component of the modeled system. The value of a variable describes the actual state of the designated component. Each variable has one of two possible values – *false* or *true* [14]. These two states are a rough approximation, however sufficient to describe the qualitative behavior of an investigated system. Even if not named Boolean, biologists routinely classify in such a binary manner. For instance, a gene is either expressed or not, and a pathway is categorized as being activated or repressed [15]. Furthermore, concentration levels in many regulatory processes behave according to a Hill-function [16], [17]. For many values of the Hill-Coefficient, this curve is sigmoidal and can be approximated by a dichotomous step-function [16], [18].

In recent years, a subfamily of Boolean networks emerged from control theory. In so-called Boolean control networks (BCN), the set of variables Xis redefined and subdivided into three categories: (1) a set of input nodes Y. A node in this set is not regulated by other components of the system. (2) a set of output nodes U. This set comprises components which are not regulating other components of the system, and (3) the inner components X. All components in this set have a regulatory effect on other components and are regulated by other components as well [19].

BNs can be considered as a directed graph. Each regulatory component is represented by one node of the graph. The directed edges between these components represent their regulatory interactions. These regulatory dependencies between the different components of the modeled system are expressed by Boolean functions. The value of each variable is determined by these Boolean functions. The state of a BN at one point in time *t* is defined by a vector $\vec{x}\left(t\right)=\left(x_{1}\left(t\right),\cdots ,x_{n}\left(t\right)\right)$. Considering all possible combinations of assignments to the *n* component this leads to a total number of $2^{n}$ possible states in the network.

In BN models, time is considered as discrete, meaning that, at each discrete *t* time, a new state of the network is updated by applying the defined Boolean functions [20].

The transition of one variable from one point in time to the next $x_{i}\left(t\right)\mapsto x_{i}\left(t+1\right)$ is done by a corresponding Boolean function $x_{i}\left(t+1\right)=f_{i}\left(\vec{x}\left(t\right)\right),\;\;f_{i}:\;B^{n}\to B$ [21].

### 2.1 Updating schemes of Boolean network models

**Fig. 2:**
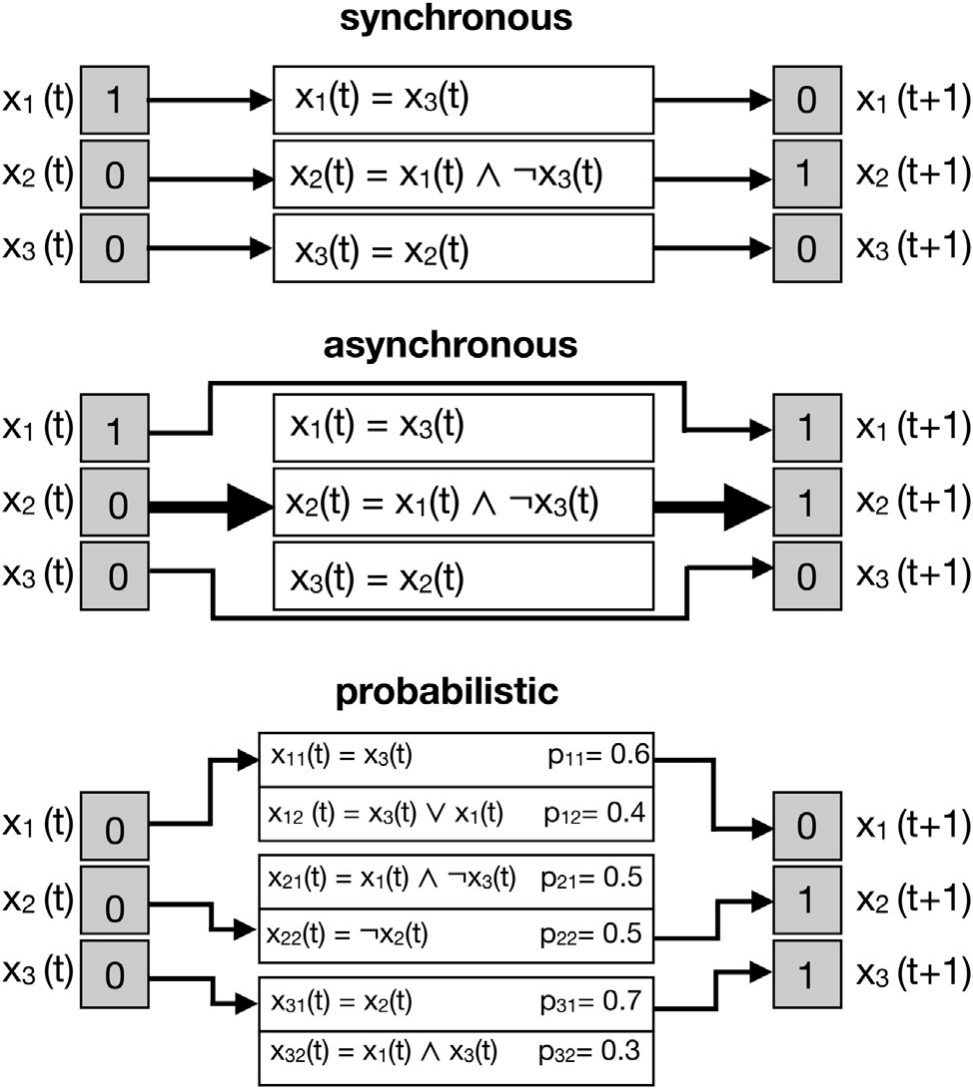
Paradigms of state transition in Boolean network models. There are three major paradigms of state transition from state x(t) to its successor state x(t + 1). In synchronous models all Boolean functions are applied at the same time while in asynchronous models only one randomly chosen function (fat arrow) is updated per step. Probabilistic models can have multiple functions with a predefined probability. One function per variable x_i_ is randomly chosen in each time step and then synchronous updating is performed.

There are three major paradigms of how BNs transit from one state to its successor (Fig. 2). When using synchronous updates, each Boolean function is applied to compute a state transition from t to t+1. The underlying assumption is that all components of the system take an equivalent amount of time to change their value [22]. Consequently, the dynamics of the BN are deterministic, and each state of the BN has one successor.

The asynchronous update paradigm assumes that only one random component is updated at each single time step [22]. This update mechanism is stochastic and leads to n possible successor states of each state, depending on the selected component. Asynchronous updating was thought to be more representative for biological systems. However, due to one single update per transition, matching the timing of the model to the real biological system leads to unrealistic durations of biological processes. For instance, if the process requires minutes in a real system, a set of downstream regulated genes may not be updated for days according to the asynchronous simulation. Furthermore, it should be kept in mind that biological processes depend on each other, e.g. a protein can never be active without being previously transcribed. Besides discussions on realistic representation of biological timings, it should be underlined that studies considering different variants of asynchronous updating revealed that synchronous updating may be more relevant for evaluating robustness of the system. In this perspective, synchronous as well as asynchronous updating lead to the same stable biological meaningful dynamic behavior [23], [24], [25], [26]. Moreover, when dealing with large BN, the run time for asynchronous simulation can become a strong limitation [22]. On these grounds, there are several sub-classes of BNs, aiming to bridge the gap between these different update strategies. The temporal BN extension allows modeling on different interactions and time scales while maintaining the deterministic nature of synchronous BNs [27]. Furthermore, a variety of different update strategies for asynchronous BNs [28] aim to limit the burst of different dynamics emerging from the asynchronous paradigm e.g. random order asynchronous or deterministic asynchronous updating [22], [23].

Probabilistic BNs allow for alternative Boolean functions for each component (each with a certain probability). The update mechanism is synchronous, and the Boolean function of each component is drawn according to its probability before each state transition. This class of BNs was introduced to incorporate the uncertainty in gene expression data [29], [30].

## 3 Properties of Boolean network models

Biological systems have some dominant patterns regarding their topology and dynamic behavior. These properties can also be observed in BN models of these systems.

### 3.1 Static characteristics

The regulatory dependencies inside biological systems form a static interaction graph with typical properties. The topology of a Boolean network emerges from the interaction of its components. There are various types of different topologies. The first Boolean networks which were analyzed had a random topology, as the networks interactions were created randomly [8]. Furthermore, often biological networks are organized in modules [31]. Modules are sets of genes which are strongly interconnected, and their function is separable from genes of other modules [31]. A modular network topology is well organized and promotes stability and evolvability at the same time [32]. Studies revealed that a variety of biological networks also exhibit a scale-free topology [33]. Gene regulatory networks, metabolic networks, and protein interaction networks show this kind of topology [34], [35], [36], [37]. Within scale-free topology, the degree of regulatory connections follows the power law distribution ($P\left(x\right)\propto x^{-\alpha }$). Fundamental properties of scale-free networks are that most of the networks’ components are lowly connected, while some of them, called “hubs”, are highly connected [33], [38]. This topology has significant effects on the robustness of a modeled system [38] making it resistant against accidental failures [39]. Regulatory components in biology usually act either as activator or inhibitor inside one particular context. Increasing concentration of an activator will lead to an increased but never decreased concentration of the target and vice-versa for repressors [40], [41]. Consequently, regulatory functions in biological networks are monotone or at least close to monotonicity [42]. Monotonicity of regulatory mechanisms can also be investigated for Boolean functions [40]. Additionally, there are other related classes of Boolean regulatory functions, such as canalyzing functions [40]. Canalyzing functions have at least one input such that for at least one input value, the output value is fixed [43]. Nested canalyzing functions are an extension of this concept where multiple variables dominate a function [44], e.g. A OR (B AND C). In this function, A = 1 dominates the assignment of the other variables. If A = 0, the second part of the function is dominated by B = 0 or C = 0, exhibiting a nested canalyzing behavior. It could be shown that biological regulation behaves similarly to canalyzing functions [45], [46], [47], leading to more robust networks compared to random regulatory functions [43], [48] and also to networks which are more robust to perturbations [49].

Analysis and investigation of these properties can give insights into the complex function of regulatory systems in biology. Studying the topology and constitution of modeled functions may reveal vulnerabilities in these robust systems or their degree of redundancy [50]. Findings like these, allow hypothesizing about the origins of certain diseases or druggable candidates.

### 3.2 Dynamic characteristics

#### 3.2.1 State graph

**Fig. 3:**
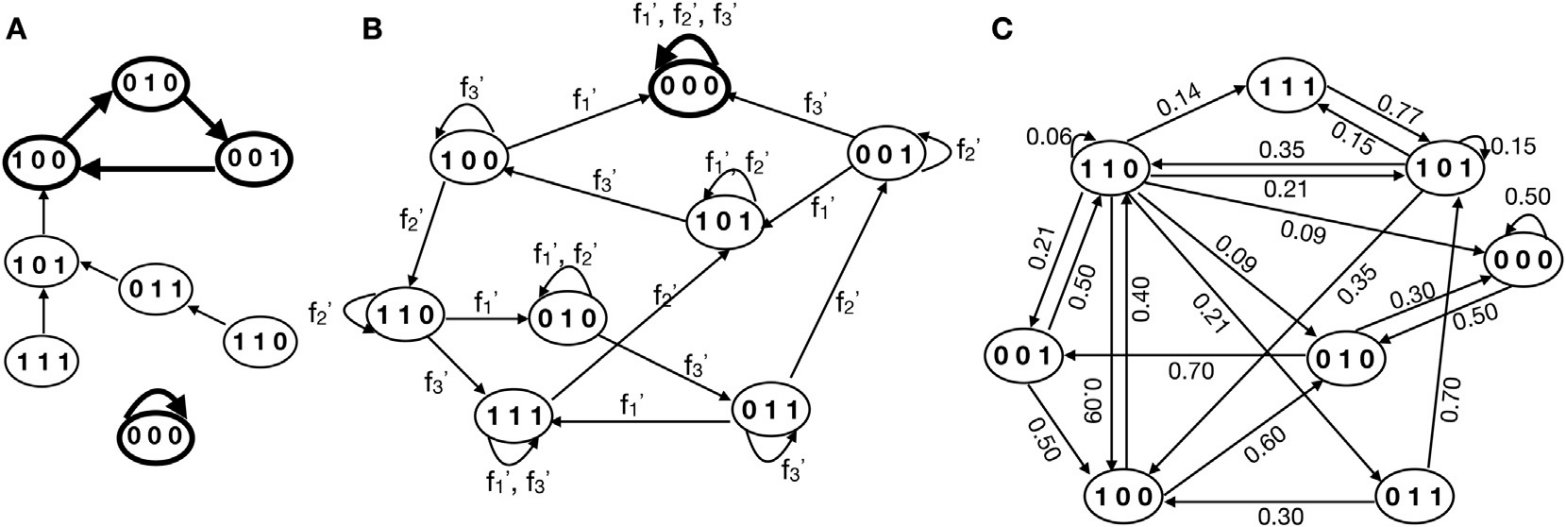
State graphs. The dynamics of Boolean network models can be depicted in state graphs showing the transition (arrows) between states (circles with activity of each component) and the progression towards attractors (bold cycled states). Here, state graphs of three interacting compounds are shown, the three-digit binary number shows the state of the network. Attractors are single states or a reoccurring sequence of states that describe the long-term behavior of the model. States that have no successor state are called Garden-of-Eden states. By synchronous updating (A) each state has a unique successor state. This is no more the case by asynchronous (B) or probabilistic (C) updating of Boolean functions. Here, updating functions (f1′, f2′, f3′) or probabilities are shown above the state transition [29].

Following the idea of moving from a static network picture to a dynamic one, the first point to assume is the implementation of time. As for the static interactions, the dynamics can be represented in directed state graphs (Fig. 3). Here, each node corresponds to one state of the network, while edges represent transitions from one state to its successor. The update strategy of the underlying BNs affects the constitution of the resulting state graph strongly (Fig. 3). In synchronous BNs, each state has only one outgoing interconnection and consequently only one possible successor state (Fig. 3A).

When using the asynchronous update strategy, the state graph becomes more complex. Each node of the graph has up to n different outgoing edges from each state, depending on the node which is selected for an update [16], [51], [52] (Fig. 3B).

Within the probabilistic scheme, a node in the state graph potentially has multiple outgoing edges, which depends on the different combinations of transition functions selected. However, a probabilistic BN behaves like a synchronous BN until the network function is changed due to a varied selection of transition functions. This leads to a switch from the state graph of one synchronous BN to another [53] (Fig. 3C).

#### 3.2.2 Long-term behavior

A trajectory through the state graph always describes the networks’ behavior over time [16], [18], [21]. State graphs contain periodic sequences of states, called attractors. Once reached, they cannot be left unless an external perturbation occurs [8], [20]. Attractors represent the long-term behavior of BNs and have been linked to biological phenotypes, making them a crucial point of interest in the analysis of BNs [18], [20], [54]. They can be subdivided into different classes. Steady-state attractors comprise only one state. These attractors occur in both synchronous and asynchronous BNs [22]. Additionally, there are attractors with more than one state. Synchronous networks may also exhibit simple cycles. Simple cycles are attractors which are formed by a sequence of states of a certain length which are periodically repeated [8], [16].

The asynchronous update strategy reveals the so-called complex attractors. Complex attractors are formed by overlapping loops which origin from the possibility of reaching more than one successor state in the asynchronous update scheme [16], [21], [51], [52]. For the one special case with an asynchronous network of size n = 1, the system cycles between 0 and 1, complex attractors and simple cycle are the same.

Probabilistic BNs allow the same attractor constructs as synchronous BNs. However, depending on the selected set of transition functions the attractors might be instable. For this reason, the dynamics of probabilistic BNs do not necessarily contain attractors [29].

#### 3.2.3 Basin of attraction

The basin of attraction comprises all states which lead to a corresponding attractor. Thus, the larger the basin of attraction is the more the attractor is likely to be biologically meaningful [24]. Furthermore, from a wet-lab point of view, the analysis of basins of attraction allows hypothesizing about the underlying decision process in the modeled regulatory system [55]. In synchronous networks, each state will eventually end up in only one attractor [8], [16], [21]. Consequently, the basins of attraction are disjunct and cannot overlap.

Due to the non-deterministic nature of asynchronous BNs individual states may reach multiple attractors depending on the selected successor states, and basins of attraction are not that well-defined. Klarner et al. [55] distinguish between strong and weak basins of attraction in asynchronous BNs. States which belong to a strong basin of attraction lead to only one possible attractor. In contrast, states in the weak basin of attractor may lead to a certain attractor but also another one.

#### 3.2.4 Spreading of information

Regulatory systems can be seen as information processing units. Each regulatory system is capable of processing a certain amount of information. Consequently, information-theoretic measures are frequent tools to study the regulatory mechanisms inside BNs.

The complexity of the information, that a system can process highly depends on the partitioning of the state graph [56]. Entropy [56] was introduced as a measure of uncertainty about the dynamic behavior of BNs. The higher the entropy, the more information is required to determine the future behavior of the network [56]. Mutual information is used in BNs [57] to measure the propagation of information through the regulatory network [58]. In the REVEAL algorithm, this measure is used to reconstruct BNs from time-series of biological data [59]. In another study, it was shown that canalyzing functions maximize the mutual information in Boolean networks [60].

## 4 Modeling Boolean networks

### 4.1 Literature based modeling

One approach to model Boolean networks is based on user knowledge and literature research. This bottom-up approach usually starts with the selection of components which are critical to describing the system of interest. Boolean network models allow for integrating different biological components and even non-physical components such as complete processes or other events. The definition of interactions between these components relies on literature research. For modeling interactions between several elements of the system, it is required to specify Boolean functions that represent the given interaction. Typically, an expert extracts natural language statements that explain specific interactions from literature which are then manually transferred to Boolean functions [61]. Logical connectives among interaction partners can be used to estimate these functions. Different tools have been developed to support the final network construction (Table 1).

**Table 1:** BN analysis tools. Summary of available tools and their scopes.

Construction	Dynamic properties	Static properties	Interventions	Tool	Main features	Construction	Dynamic properties	Static properties	Interventions	Tool	Main features
	X			ChemChains [90]	– implemented in C++ – command-line driven simulation – synchronous updating, asynchronous updating for user-selected nodes	X	X			Polynome [99]	– web service – reverse-engineering of Boolean network models from experimental time series – attractor search – parameter estimation for continuous models
	X			SimBoolNet [92]	– Java plugin for Cytoscape – graphical interface – visualization of dynamic changes	X	X		X	SQUAD & BoolSim [100]	– graphical interface – conversion of logical into continuous models – attractor identification (stable states and cyclic attractors) – continuous simulation – perturbation experiments
	X			MaBoSS[95]	– C++ implementation – simulation of continuous time Markov processes based on BN – definition of transition rates and time trajectories – evolution of probabilities over time is estimated	X	X		X	ViSiBooL [27], [91]	– graphical interface – construction and analysis of synchronous and asynchronous BN-visualization of attractors – extension: intervention screening
	X			Pint [96]	– command line or Phython tool – very large-scale networks including BN and multi-valued networks ranging – lists fixed points, successive reachability properties – cut sets and mutations for reachability – model reduction preserving transient dynamics		X	X	X	CellNetAnalyzer [94]	– Matlab tool – graphical interface – BN, multivalued logic, ODE models – stoichiometric and constraint-based formalization and analysis, interaction graphs, steady state analysis – computation of minimal intervention sets
X	X			The Cell Collective [97]	– web based platform implemented in Java – graphical interface – probability of being active can be assigned to external regulators – platform to distribute published BN	X	X	X		GINsim [93]	– JAVA application – graphical interface – multi-valued logical models-state transition graphs for synchronous and asynchronous updating – determination of stable states
X	X			CellNOpt [98]	– for R, Matlab, Python and Cytoscape – graphical interface for Cytoscape (plugin CytoCopter) – creation of logic-based models (BN, Fuzzy or ODE) based on prior knowledge and training against experimental data – extension CNORdt allows to train a BN against time-courses of data	X	X	X	X	BoolNet [85]	– R-package – construction and analysis of synchronous, asynchronous, probabilistic and temporal BN – reverse-engineering form time series – simulation and visualization of attractors, transition graphs and basins of attractions – robustness analysis and comparison to randomly generated networks
X	X			BooleanNet [15]	– Python source code – synchronous, asynchronous, ranked asynchronous, time synchronous and piece wise differential updating – extension to hybrid models by piecewise differential equations	X	X	X	X	PyBoolNet [101]	– Python tool – construction, visualization, analysis and manipulation of large networks – synchronous, asynchronous and mixed updating – execution of graph algorithms and graph drawing – several attractor analysis and basin of attraction – model checking tool

### 4.2 Data-driven modeling

Alternatively, data-driven top-down approaches can be applied. Numerous approaches to reconstruct Boolean networks have been published. In most cases binarization of high-throughput data e.g. gene expression is required in order to infer the Boolean functions [62], [63]. Reconstruction algorithms are then used to predict regulatory interactions [40], [41] or complete Boolean functions [64].

### 4.3 Random Boolean networks

In contrast to models which represent specific regulatory systems of interest, random Boolean networks are one helpful tool, which allows investigating a variety of different dynamic properties of BNs and regulatory systems. Random BNs are typically based on three different parameters n,k,p [65], [66]. The parameter n defines the number of components in the system. k represents the number of inputs of each regulatory function, and p indicates the probability of a regulatory function to return 1 [16], [65]. Network nodes, regulatory interactions, and the underlying Boolean functions are then generated randomly according to these parameters. Classic random Boolean networks are updated synchronously [65]. Random Boolean networks are useful tools to investigate general concepts of regulatory mechanisms. Then, the latter can be applied to specific biological contexts. Kauffman and others applied this concept to the yeast cell cycle model [48], [67], [68], [69], [70], [71]. Further extensions of the random BNs paradigm are given by Darabos and Tommasini [72] and Graudenzi et al. [73]. They used a semi-synchronous update scheme and studied the influence of “memory”, i.e. decay time of gene products, robustness and impact of perturbations in random BN systems.

### 4.4 Ensemble approach

Another approach to model Boolean networks is the ensemble approach. Contrasting the modeling and investigation of a particular set of regulatory components and their dynamics, ensembles are used to study generic properties of biological systems. In the ensemble approach, sets of BNs which match to properties of real systems are used to gain new insights into the dynamics of real systems [74]. Originally, an ensemble of networks is sampled from all possible random BNs with a certain assignment for n,k. All these BNs in the ensemble have certain properties of interest which match the real system [75]. This ensemble can then help to understand the organizing principles which explain the generic behaviors of its members [76]. This approach has been used to analyze the stability of the yeast transcriptional network [48] and the ordered behavior of hierarchical canalyzing functions [45].

### 4.5 From theory to model

Up to this point, different BN modeling approaches have been presented. However, the question that still arises is how to actually translate all this theoretical information into a model. The first task is to clarify the biological question behind the modeling approach. If the investigator is interested in general properties, such as topological features that confer certain behaviors, then random BNs and/or the ensemble approach are suitable modeling strategies.

If one is interested in investigating a certain biological pathway or set of pathways one would have to check how much information is available on the biological phenomena of interest. Starting with literature-based modeling is definitely a first approach, as existing knowledge needs to be included in the model. If additionally measurement data in the form of time-series is available one could augment the initial models with the data driven reconstruction. Note that this strategy does not necessarily reveal a single model variant [77]. Multiple regulatory interactions are possible to recapitulate the given dynamics and not all interactions may represent biological reality. However, also the sole construction of literature-based models might have some critical points to consider. The first challenge is the construction of the model itself and, in particular, of the Boolean functions. Assuming the impact of canalyzing functions in biological networks [45], [46], [47], Boolean functions can be modeled using a certain pattern: multiple activators are supposed to have equal contribution and are thus connected by the OR-operator. Conversely, inhibitors are connected using the AND-operator. Therefore, the node of interest will be active only when at least one of the activators is present and none of the inhibitors is active [78]. From this simple basic construct, Boolean functions can become more and more complex depending on the specific cases.

Moreover, not always there is only one unique function to describe a specific interaction. In this case the investigator can try different options and choose the best representing the final behavior of the real biological system [78]. Also, in the case that independent possible functions are identified, a probabilistic updating scheme is suggested [29], [30]. Instead, if only one final Boolean function is present for each node the most suitable update schemes are synchronous or asynchronous ones. Again, the decision between one or the other may be guided by the biological question itself. For example, differentiation is a continuous decision-making process towards one lineage or the other starting from stem cells, to progenitors until differentiated cells. Therefore, an approach that allows multiple trajectories within the state space could give a good representation of the biological scenario. Based on this idea, Krumsiek et al. modeled hierarchical differentiation of myeloid progenitors by means of asynchronous Boolean network models [79].

To sum up, it is possible to apply different modeling approaches and knowledge in BN models to tackle different biological questions. Once a model is constructed with a desired modeling approach, simulation and analysis of the dynamic behavior of the system can be performed.

## 5 Simulation and analysis of Boolean network models

The simulation of mathematical models allows investigating the long-term behavior of a system in a cost-efficient manner [7], [80], [81]. These models can be used to recapitulate normal behavior or to generate hypotheses about the impact of interventions. Promising model simulations can later be studied in laboratory experiments [13], [80].

Based on the concept of BN models, investigating long-term behavior is associated to search for attractors and analysis of the transition or path towards an attractor. Various analysis methods are available for this purpose. One can either search for all attractors of the network [10], [12], or only for special attractors, which result from a known initial state [11], [13]. However, the identification of all attractors by exhaustively calculating all $2^{n}$ successor states of a network is computationally demanding and could be shown to be NP-hard [82]. Already determining whether a given BN has one attractor is NP-hard, while finding on singleton attractor takes $O\left(1.587^{n}\right)$ time even for AND/OR BNs [83]. As a consequence, this procedure is only feasible for small networks [16]. Model-checking algorithms can help to determine the attractors of larger network models. The attractor search can be converted into a satisfiability problem [84] for which heuristics exist, but without revealing state transitions [84], [85]. Additionally, simulating BN models can unravel mechanistic regulations by studying transition from an initial state towards the attractor of interest [13].

A recent approach aims at converting Boolean control networks (BCN) into discrete time bi-linear systems. This allows to investigate the dynamics of Boolean control networks using standard matrix analysis [86]. The semi-tensor product of matrices [87] was introduced as new matrix product and enables to express a BCN in algebraic form [88]. This approach can shed light on various challenges. Stability problems, controllability, and fault detection could be investigated using the semi-tensor-product. However, as for other BN approaches, the exponential time complexity limits semi-tensor-product approaches to focus on networks with a relatively small number of components [89].

Multiple toolboxes have been developed to simulate and visualize the dynamics of BN models. While tools like BoolNet [85], BooleanNet [15] or ChemChains [90] require some programming skills, there are also tools such as ViSiBooL [27], [91], SimBoolNet [92], GINsim [93] or CellNetAnalyzer [94] that use a graphical user interface (for a more detailed description see Table 1).

### 5.1 Identification of biologically meaningful attractors

A characteristic of BN models is that the number of attractors is relatively small in comparison to their state space [25]. Due to the finite state space and their deterministic nature, BN models with synchronous updating scheme have at least one attractor [102]. Indeed, many BNs have more attractors, and not all may be biologically meaningful [81]. For instance, attractors with small basins of attractions are considered to be less relevant [25], [102]. Based on this assumption, the basin of attraction can be an indicator of biological relevance [11], [102].

Moreover, the stability of attractors is one vital property to interpret their biological plausibility and relevance. Stability analysis of attractors in synchronous Boolean networks showed that many of them are artefacts from the synchronous update scheme [24]. The remaining attractors, in contrast, are stable towards delays and other noise [103]. Attractors which are stable towards internal noise such that state fluctuations caused by that noise are not sufficient to make the system change are called ergodic state sets [103], [104], [105]. Since biological systems can be viewed as being persistently perturbed, ergodic attractors were proposed by Kauffman as the subset of attractors connected to cellular phenotypes [103]. In particular, ergodic sets have been applied to study the role of noise during cellular differentiation and cell reprogramming [103], [105].

### 5.2 Robustness analysis

**Fig. 4:**
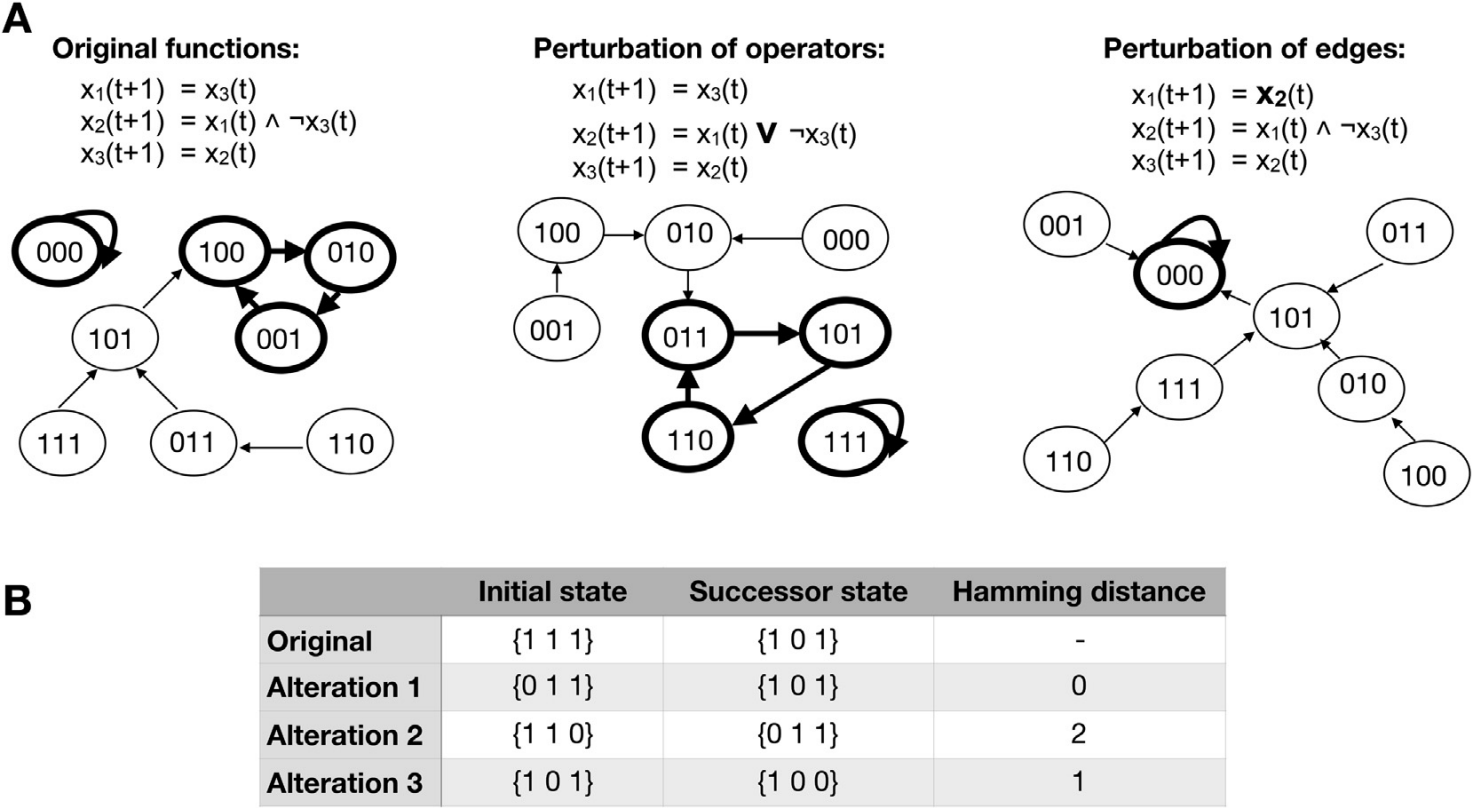
Perturbation of Boolean network models. Boolean network model can be altered on a structural level (A) by changing operators or edges (thick arrows). These alterations can change transitions between states (arrows) and can lead to different attractors (thick cycles in the state graphs). Thus, comparing the number of same attractors between the original and the perturbed network can be used as readout. Additionally, state changes by bit flipping can perturb Boolean network models (B). Comparing the sequences of the successor states of the original and the perturbed network can be used as readout. The Hamming distance counts all alterations of elements between the two sequences.

Biological systems are exposed to environmental changes or genetic mutations that might result in physiological changes [1]. These alterations are comparable to state changes or structural modifications [102]. Nevertheless, biological systems are relatively impervious against random variations [1], [106]. Reasons for these robustness properties are feedback regulations and the redundancies of nodes [1], [106], [107], which lead to the previously described scale-free topology. Therefore, robustness measurements can be taken into account for the identification of biologically meaningful attractors or networks, as suggested by Kauffman [20], [81]. In total, there are two standard classes of perturbations in BN models. They can be perturbed on a structural level or a dynamical level (Fig. 4) [108].

Structural alterations can be changes of inputs or operators in the regulatory functions. Exemplarily, the removal of canalyzing functions by perturbation can lead to a loss of robustness [48]. A prominent structural disruption, applied in different studies of BNs [11], [13], [77], is fixing a compound of the system to either *false* or *true.* This kind of perturbation corresponds to *in silico* knock-out or overexpression experiments [22]. All these perturbations may change the structure of the state graph and can lead to an altered attractor landscape (Fig. 4A). Therefore, a comparison of attractors between the original and the perturbed network can be considered as a readout to determine the effect of the applied perturbation. This kind of simulation gives insights into the regulatory mechanisms of the modeled system.

In contrast to structural changes, dynamic alterations do not affect the underlying BN functionality. One kind of perturbation is to apply bit-flip mutations that may perturb the system temporally (Fig. 3B). Usually, some bits in the bit string which define a particular state of the network are flipped at random. This perturbation corresponds to a switch from one node in the state graph to another. However, the structure of the state graph and, thus, the attractor landscape is kept intact. Depending on the robustness of the network, this kind of perturbation may have only temporal effects. If the perturbed state is still part of the same basin of attraction as the original state, there are no long-term effects. However, path lengths and state transition towards the attractor may change [77]. Alternatively, the applied bit-flip may also lead to different long-term outcomes. In this case, the applied temporary perturbation leads to a switch from one basin of attraction to another. The resulting effects can be quantified by comparing the original trajectory and the trajectory after perturbation. A measure that can be applied for this purpose is the Hamming distance, as suggested by Gershenson et al. [107]. The smaller the effect of the applied perturbation, the lower is the Hamming distance between the two trajectories. A lower Hamming distance can indicate a more robust network [77].

**Fig. 5:**
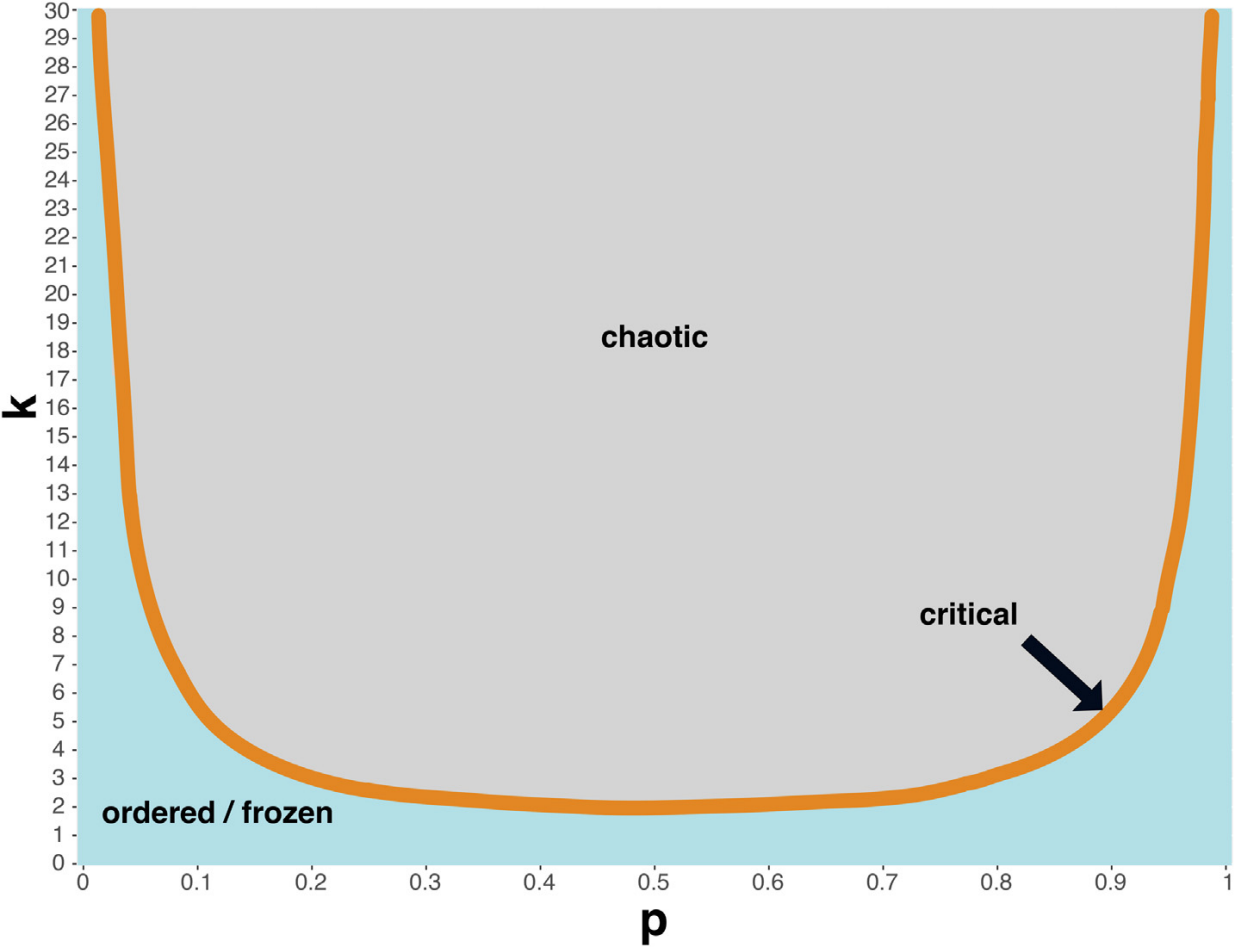
Regimes of random Boolean networks. The three different regimes of random Boolean networks depending on the two parameters *k* and *p*. If 2∙k∙p∙(1-p)>1 the networks belong to the critical regime, if <1 to the ordered regime. Networks in between are in the critical regime or at the “edge of chaos”. For p=0.5 networks with k=2 are considered to be at the “edge of chaos”.

Already in the first works of Kauffman [20], [109] and carried on by others [110], [111] it was stated that random BN models could be found in different regimes, e.g., ordered/frozen, chaotic, and critical state. In this landscape, living systems have been placed at the so-called “edge of chaos” [20], [104], [105], [109], [112], [113]. In this regime, a network is stable enough to keep information but also flexible enough to explore the state space and spread perturbations to allow evolvability [65]. The simultaneous property of stability and evolvability in biological networks was also confirmed by Aldana et al. by studying the effects of gene duplication on the attractor landscape [114]. Various approaches using Hamming distance such as Derrida plots [115], [116], [117] and Lyapunov exponent [118], [119], [120] can be used to investigate the spread of damage through the network. In particular, Derrida proposed a numerical analysis of Kaufmann’s simulation, inferring the critical regimes for random networks with different p parameters, introduced in the previous sections. In general, the more extreme the p parameter is, the more the system resides in a frozen regime [115], [116], [117], [119], [120], [121]. Vice versa chaotic regimes arise in networks with lower p parameter (Fig. 5). Moreover, k for critical regimes can be expressed in function of p $\left(2\cdot k\cdot p\cdot \left(1-p\right)\right)$. In Kaufmann’s random networks, p is equal to 0.5, which describes so-called unbiased functions. In this case, as shown by Kaufmann’s simulations, the k of the critical regime can be calculated and is equal to 2 [115], [116], [117], [119], [120], [121]. Derrida’s analysis has then been applied to study the spread of perturbation characteristic of networks with different regimes giving the final observation that in frozen regimes there will be a minimal spread of noise and in chaotic ones maximal. This information can be represented in Derrida plots, where Hamming distances at t and t + 1 time points are shown [115], [122].

### 5.3 Identification of intervention targets

Robustness of a system is a double-edged sword [1]. Cancer cells, for example, acquire a high degree of robustness against induction of apoptosis [123]. Therefore, the identification of promising intervention targets that change the dynamics of degenerated cells is of special interest. In this direction several modeling approaches have been proposed to suggest new therapeutic target candidates [13], [124], [125], [126], [127], [128], [129], [130], [131]. Moreover, even if single targets are identified, they may result in ineffective long-term inhibition due to insurgence of resistances [132], [133], [134], [135]. For this reason, selections of combined therapeutic approaches become of central interest. In this perspective, dynamic analyses of BN can provide suggestions for promising candidates, avoiding expensive and long inhibitor screenings [136]. Exemplarily, Flobak et al. set up a model to predict drug synergies in gastric cancer cells. Here, they integrated pathway knowledge from databases and literature known to be involved in gastric cancer. Each node state in the resulting attractors was then calibrated against baseline markers in gastric cancer cells. The established model was afterwards used for perturbation screening on seven known inhibitors available for targeting cancer cells, and all possible combinations were screened *in silico*. Thereby they identified two new synergistic targets that were further successfully tested in cell assays showing the prediction power of *in silico* approaches in preclinical studies [136]. Different tools and algorithms are available to perform these alteration screenings (Table 1).

## 6 Conclusion

Traditionally, life science applies a reductionistic approach and focuses on a single compound and its effect in a specific process or pathway of interest [137], [138]. This approach has successfully identified many components, their interactions, and the underlying molecular mechanisms [139]. However, it does not describe the properties of a complete system which emerges from the interactions of its components. Mathematical models of biological systems, in contrast, can be applied to close this gap and guide laboratory experiments. Multiple kinds of models vary in their complexity. The choice of the right model depends mainly on the availability of the required information. BN models are attractive models to study the complex dynamic behavior of processes with limited knowledge. The underlying regulatory functions to model a BN can usually be extracted directly from natural language statements in the literature. No further quantitative information, such as kinetic parameters is required.

Despite this rough approximation, BNs proved to be valid tools to simulate the qualitative behavior of a modeled system adequately.

Synchronous BNs have a deterministic nature which is suitable for interpretation, but the synchronous update is a rough approximation of the different temporally regulated mechanisms in a system. Asynchronous BNs introduce another degree of freedom by allowing for modeling on regulatory interactions on different time scales. However, the stochastic nature of this update paradigm leads to complicated and hard to interpret dynamics with biologically irrelevant state transitions [140]. Probabilistic BNs introduced an update strategy which can cope with uncertainty in regulatory mechanisms. This type of model heavily relies on the estimated probabilities and thus may be prone to errors if these parameters are not estimated correctly. Additionally, there is a variety of different sub-classes of paradigms available.

In summary, the type of update strategy depends on a user’s intentions and the available information.

Besides, random BN models have been proven to be a valuable tool for investigating general properties of biological networks, such as topologies that confer robustness.

For each of those paradigms, there are numerous algorithms and tools available which allow simulating the dynamics of a modeled regulatory system. Each of these approaches aims to give insights into the mechanics and dynamic structure of a network of interest. In this review, we summarized different properties of BN models and their importance for a better understanding of a system under investigation. Additionally, we named several tools, which are available for BN modeling and simulation.

## 7 Author statement

HAK and MK conceptualized the manuscript and provided funding. The original draft was written and further revised by JDS, SDK, NI, MK and HAK. Figures and tables were created by JDS, SDK and NI.

## Declaration of Competing Interest

The authors declare that they have no known competing financial interests or personal relationships that could have appeared to influence the work reported in this paper.
